# Human circulating basophils lack the features of professional antigen presenting cells

**DOI:** 10.1186/1479-5876-10-S3-P4

**Published:** 2012-11-28

**Authors:** Meenu Sharma, Pushpa Hegde, Vishukumar Aimanianda, Jean-Paul Latgé, Srini V Kaveri, Jagadeesh Bayry

**Affiliations:** 1 Institut National de la Santé et de la Recherche Médicale, Unité 872, Paris, France; 2 Universite de Technologie de Compiègne, Compiègne, France; 3 Unité des Aspergillus, Institut Pasteur, Paris, France; 4 Centre de Recherche des Cordeliers, Equipe 16- Immunopathology and therapeutic immunointervention, Université Pierre et Marie Curie – Paris 6, UMR S 872, Paris, France; 5 Université Paris Descartes, UMR S 872, Paris Université Pierre et Marie Curie – Paris 6, UMR S 872, Paris, France

## Background

Professional antigen presenting cells (APC) are those which can uptake the antigens, process and present them in the context of MHC molecules and co-stimulatory molecules to CD4^+^ T cells leading to activation and polarization of CD4 T cell responses. Recent reports in mouse have shown that basophils can function as APC. Murine basophils express MHC class II and co-stimulatory molecules CD80 and CD86, can capture IgE-antigen complex or soluble antigens leading to presentation of antigens, secretion of IL-4 and polarization of Th2 responses. Therefore, we explored whether human circulating basophils do possess features of professional APC.

## Materials and methods

Basophils from basophil-rich peripheral blood mononuclear cells (PBMC) were isolated by using basophil isolation kit II (Miltenyi Biotech). Dendritic cells (DC) were generated by culturing CD14^+^ monocytes in GM-CSF and IL-4 for five days. The APC were activated by stimulation with Aspf1 protein of *Aspergillus fumigatus*. T cell polarization was determined by co-culturing Aspf1-primed DC or basophils with autologous CD4^+^ T cells.

## Results

We found that unlike monocytes and DC, steady-state circulating human basophils do not express HLA-DR and co-stimulatory molecules CD80 and CD86. Basophils remained negative for these molecules even after stimulation with soluble antigen Aspf1 indicating that although basophils from mice show the expression of antigen presenting and co-stimulatory molecules, human basophils from circulation do not express these molecules even when stimulated with soluble antigens. Furthermore, DC that were primed with Aspf1 increased the production of IL-4 in CD4+ T cells thus indicating the promotion of Th2 responses by Aspf1-primed DC. However, Aspf1-pulsed basophils did not potentiate Th2 responses.

## Conclusions

These results demonstrate the inability of circulating human basophils to function as professional APCs. As presence of IgE-reacting to Aspf1 is a common feature of allergic broncho-pulmonary aspergillosis (ABPA) and allergic asthma, based on our results one may conclude that IgE antibody responses to Aspf1 in patients are mediated mainly via DC and not by basophils.

